# Oral anticoagulation discontinuation after atrial fibrillation ablation: a systematic review and meta-analysis of randomized trials

**DOI:** 10.1093/ehjopen/oeag067

**Published:** 2026-05-22

**Authors:** Ahmed Salih, Antonio Creta, Hussam Ali, Riccardo Cappato, Rui Providencia

**Affiliations:** School of Medicine, Imperial College London, Imperial College Faculty of Medicine, Faculty Building, South Kensington Campus, London SW7 2AZ, UK; Institute of Health Informatics Research, University College London, 222 Euston Road, London NW1 2DA, UK; Barts Heart Centre, St Bartholomew's Hospital, West Smithfield, London EC1A 7BE, UK; Arrhythmia and Clinical Electrophysiology Center, IRCCS, MultiMedica Group, Via Milanese, 300, 20099 Sesto San Giovanni, Milan, Italy; Arrhythmia and Clinical Electrophysiology Center, IRCCS, MultiMedica Group, Via Milanese, 300, 20099 Sesto San Giovanni, Milan, Italy; Institute of Health Informatics Research, University College London, 222 Euston Road, London NW1 2DA, UK; Barts Heart Centre, St Bartholomew's Hospital, West Smithfield, London EC1A 7BE, UK

**Keywords:** Atrial fibrillation, Catheter ablation, Oral anticoagulation, DOAC, Stroke, Major bleeding

## Abstract

**Aims:**

The need for long-term oral anticoagulation (OAC) after apparently successful atrial fibrillation (AF) catheter ablation remains uncertain. Although ablation reduces AF recurrence, it is unclear whether this translates into a low stroke risk to sufficiently discontinue anticoagulation. This systematic review assesses whether stopping OAC after successful AF ablation affects thromboembolic or bleeding risk.

**Methods and results:**

We searched MEDLINE, Embase, and Scopus up to 13 November 2025 for randomized controlled trials (RCTs) enrolling adults with AF who underwent catheter ablation and were subsequently randomized to continue or discontinue OAC. Outcomes were pooled using random-effects models. The primary endpoints were stroke, systemic embolism, and major bleeding. Three RCTs met inclusion criteria (*n* = 2324). Participants remained arrhythmia-free for at least 6 months before randomization and had mean CHA_2_DS_2_VASc score range of 2.0–2.6. Over a median follow-up of 25.14 months (IQR 17.44–30.57), discontinuing OAC did not increase stroke risk [OAC 0.86% vs. no OAC 0.69%; risk difference (RD) 0.24%; 95% confidence interval (CI) −0.67% to 1.15%; *P* = 0.61]. No systemic embolic events occurred in either group. Annualized stroke incidence was similarly low between groups (incidence rate ratio 1.32; 95% CI 0.32–5.41; *P* = 0.70). Continuing OAC significantly increased major bleeding (OAC 0.90% vs. no OAC 0.34%; RD 0.91%; 95% CI 0.17–1.65%; *P* = 0.02).

**Conclusion:**

In patients who remain arrhythmia-free after AF ablation, stopping OAC did not increase thromboembolic events and substantially reduced major bleeding, suggesting that in post-ablation patients with low thromboembolic risk, and no demonstration of AF relapse for at least 6–12 months, discontinuation of OAC may be potentially safe.

## Introduction

Atrial fibrillation (AF) is the most common sustained cardiac arrhythmia and a major contributor to global cardiovascular morbidity and mortality, conferring substantial risks of stroke, heart failure, and death.^1^ Catheter ablation is now an established rhythm control strategy that is superior to medical therapy in maintaining sinus rhythm and improving symptoms and quality of life.^2,3^ However, the optimal strategy for anticoagulation after ablation remains unknown.

Ablation substantially reduces AF recurrence and arrhythmia burden, but whether this reduction translates into sufficiently lower thromboembolic risk to justify discontinuation of oral anticoagulation (OAC) is still unclear. Mendelian randomization studies support a causal relationship between AF and stroke,^4^ and two recent systematic reviews of randomized controlled trials indicate that AF ablation may reduce the risk of stroke.^5,6^ Consistent with this, observational and registry data suggest that discontinuing OAC may be safe in carefully selected low-risk patients.^7^

The current European Society of Cardiology (ESC) and the American College of Cardiology/American Heart Association/Heart Rhythm Society guidelines recommend continuing OAC for at least 2–3 months after ablation in all patients and determining long-term anticoagulation mainly based in CHA_2_DS_2_VA or CHA_2_DS_2_VASc scores.^8,9^ Thus, patients who would otherwise qualify for anticoagulation are recommended to continue treatment, even if they are arrhythmia-free. Despite guideline recommendations, there remains substantial variation in practice. In a survey of authors of the 2017 AF ablation consensus, 23% reported that they would consider discontinuing OAC after successful ablation, and the consensus document itself allows OAC discontinuation (Class IIb recommendation) when guided by stroke risk, patient preference, and regular ECG monitoring.^2^

Recent randomized controlled trials have directly compared continuation vs. discontinuation of OAC after successful AF ablation. We performed a systematic review and meta-analysis of randomized trials comparing continuation vs. discontinuation of anticoagulation after AF ablation, to clarify the effects on thromboembolic events and bleeding.

## Methods

This systematic review and meta-analysis was conducted in accordance with the Preferred Reporting Items for Systematic Reviews and Meta-Analyses (PRISMA 2020) statement.^10^ The prospective meta-analysis protocol was registered on the International Prospective Register of Systematic Reviews (PROSPERO; CRD42024508688).

The research question, structured using the Population, Intervention, Comparator and Outcome (PICO) framework, was ‘In adults who remain arrhythmia-free after successful catheter ablation for atrial fibrillation, does discontinuation of long-term oral anticoagulation, compared with continued therapy, affect the risks of thromboembolism and bleeding?’

### Search strategy and study selection

We searched MEDLINE, Embase, and Scopus from inception to 14 November 2025. The search strategy combined terms related to AF, catheter ablation, and anticoagulation. Reference lists of all eligible trials and relevant reviews were screened to identify additional studies. The full search strategy is included in Supplementary material online, *Table S1*.

Two reviewers (A.S. and R.P.) independently screened titles and abstracts, followed by full texts of potentially relevant articles. Disagreements were resolved by discussion and, when necessary, adjudication by a third reviewer (A.C.). Trials were eligible if they enrolled adults (≥18 years) with a history of AF who had undergone catheter ablation and randomized participants, after an initial post-ablation period to continuation vs. discontinuation of OAC.

### Data extraction and risk of bias assessment

Two reviewers (A.S. and R.P.) independently extracted data from each included trial using a standardized form. Extracted information included study design, year and country, inclusion and exclusion criteria, ablation strategy and definition of successful ablation, baseline demographic and clinical, anticoagulant agent and dosing, timing of randomization relative to the index ablation, monitoring strategy for atrial arrhythmia recurrence, follow-up duration, and number of events for each outcome in each trial arm. Where available, person-time at risk and event rates per 100 patient-years were also abstracted.

The primary effectiveness and safety outcomes were stroke (ischaemic or haemorrhagic), systemic thromboembolism, and major bleeding (as defined by each trial, typically according to the International Society on Thrombosis and Haemostasis criteria). Secondary outcomes included intracranial haemorrhage, gastrointestinal bleeding, nonmajor bleeding, and silent stroke [defined as new ischaemic lesions detected on brain magnetic resonance imaging (MRI) in the absence of clinical neurological symptoms]. Outcomes were extracted on an intention-to-treat basis whenever possible.

Risk of bias for each outcome in each trial was assessed independently by two reviewers using the Cochrane Risk of Bias 2 (RoB 2) tool. Disagreements were resolved by consensus. The certainty of the evidence for each main outcome was subsequently appraised using the GRADE framework, taking into account risk of bias, inconsistency, indirectness, imprecision, and publication bias.^11^ For outcomes with a sufficient number of studies (defined as 10 or more), funnel plots will be used to investigate small-study effects and potential publication bias.

### Data synthesis and statistical analysis

For each dichotomous outcome, we calculated risk ratios (RRs) with 95% confidence intervals (CIs) to quantify relative treatment effects and risk differences (RDs) to estimate absolute between-group differences. When person-time data were available, incidence rate ratios (IRRs) were derived to compare annualized event rates. Risk difference was the primary method for reporting because absolute effects are more clinically meaningful in this low-event post-ablation population. Risk differences quantify the absolute difference in risk and remain stable with sparse or zero event data, whereas relative measures may appear inflated despite minimal absolute differences.^12^ Risk ratio was included as a sensitivity analysis.

Numbers needed to treat (NNT) or harm (NNH) were computed as the reciprocal of the pooled RD, with direction interpreted according to the outcome.


$$NNT/NNH=\frac{1}{Absolute\;Risk_{treatment}\;-Absolute\;Risk_{control}}$$

Random-effects meta-analyses were conducted using the Der Simonian–Laird estimator for between-study variance for the primary analyses. Statistical heterogeneity was assessed using the I^2^ statistic and τ^2^; I^2^ values of approximately 25%, 50%, and 75% were considered to represent low, moderate, and high heterogeneity, respectively. Where heterogeneity exceeded 75%, pooled estimates were interpreted with caution, and potential sources were explored qualitatively. For outcomes where a sufficient number of studies are available (defined as 10 or more), we plan to use meta-regression to explore reasons for observed heterogeneity.

Subgroup analyses were planned based on the following patient characteristics: gender, age group, race/ethnic background, BMI class, and high vs. low CHA_2_DS_2_VASc score.

All statistical analyses were performed in R (version 4.5.2; R Foundation for Statistical Computing, Vienna, Austria) using the meta packages.^13,14^ All *P*-values were two-sided, with statistical significance defined as *P* < 0.05.

## Results

**Figure 1:**
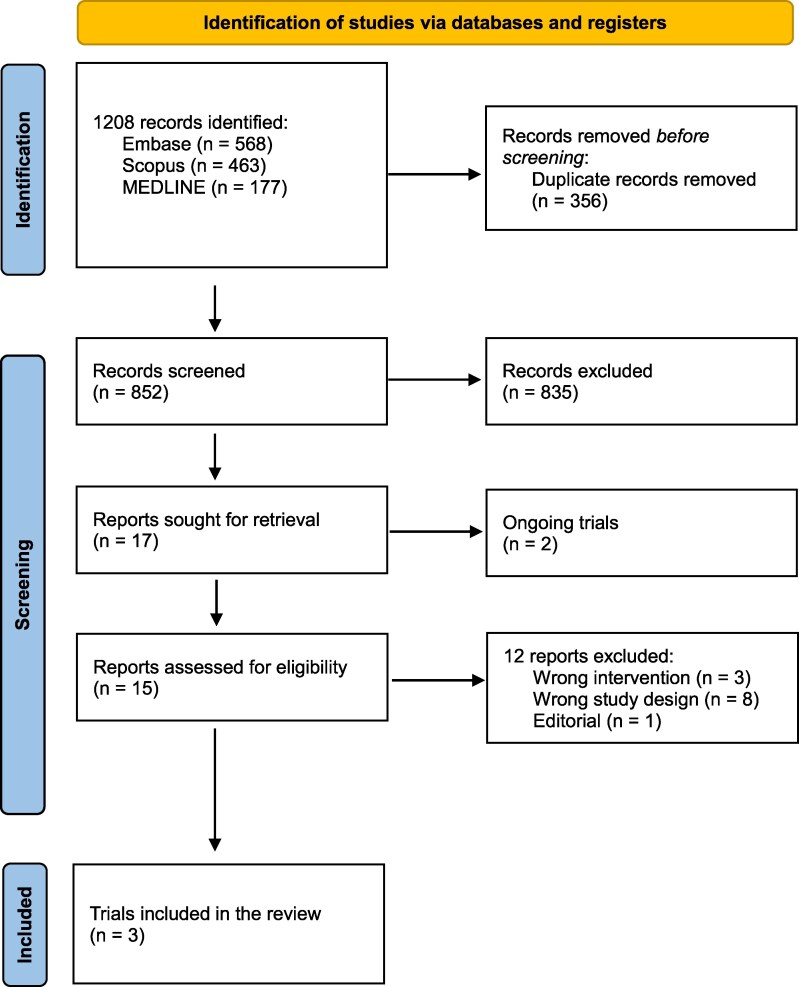
PRISMA flowchart.

The search strategy identified 852 studies, from which three completed randomized controlled trials met the inclusion criteria and were included in this systematic review^15–17^ (Figure 1). Two additional trials were identified as ongoing.^18,19^ A total of 2324 patients were included in this meta-analysis. The mean age across studies ranged from 63 to 67 years, and women accounted for 33.5% of the overall study population (*Table 1*). All trials enrolled patients who had undergone successful catheter ablation for AF and remained arrhythmia-free for at least 6 months prior to enrolment (*Table 2*). Timing from ablation to discontinuation of OAC, treatment regimens, and follow-up duration varied across the three studies.

**Table 1:** Baseline characteristics of participants in the three randomized trials

Study, year	Country	Randomis timing	Treat arm	Subj	Age (years)	Female	CHA_2_DS_2_-VASc	HAS-BLED	Parox AF	Persist AF	Long Persist AF	Prior Stroke/ TIA	HF	HTN	DM	LA Diameter (mm)	LVEF (%)
**ALONE-AF**	Japan	Median: 29M IQR 16–56M Post last ablation	DOAC	423	65 ± 8	113 (26.7%)	2 (1–3)	2 (1–3)	131 (31.0%)	292 (69.0%)	—	24 (5.7%)	62 (14.7%)	291 (68.8%)	90 (21.3%)	40 (37–44)	62 (57–66)
			No treatment	417	63 ± 8	96 (23.0%)	2 (1–3)	2 (1–3)	141 (33.8%)	276 (66.2%)	—	23 (5.5%)	66 (15.8%)	293 (70.3%)	68 (16.3%)	40 (37–43)	61 (57–66)
**OCEAN**	Multinational	Median: 16M IQR 13–25M Post last ablation	Rivaroxaban 15 mg OD	641	66.3 ± 7.1	183 (28.6%)	2.2 ± 1.1	NR	431 (67.2%)	204 (31.8%)	6 (0.9%)	28 (4.4%)	4 (0.8%)	442 (68.9%)	96 (15.0%)	40.7± 16	NR
			Aspirin 70–120 mg OD	643	66.3 ± 7.6	184 (28.6%)	2.2 ± 1.1	NR	421 (65.5%)	212 (33.0%)	10 (1.6%)	50 (7.8%)	10 (2.2%)	434 (67.5%)	68 (10.6%)	40.4 ± 19.2	NR
**ODIn-AF**	Germany	>6M Post last ablation Median: NR	Dabigatran 110–150 mg BD	99	67.3 ± 7.2	43 (43.4%)	2.6 ± 0.8	1.3 ± 0.7	65 (65.7%)	30 (30.3%)	6 (6.1%)	NR	23 (23.2%)	86 (86.9%)	21 (21.2%)	NR	58 ± 9.8
			No treatment	101	67.1 ± 7.7	45 (44.6%)	2.6 ± 7	1.5 ± 0.7	68 (67.3%)	33 (32.7%)	10 (9.9%)	NR	12 (11.9%)	89 (88.1%)	20 (19.8%)	NR	55.7 ± 8.6

Baseline demographic and clinical characteristics of participants enrolled in the ALONE-AF, OCEAN, and ODIn-AF trials, stratified by randomized treatment group (OAC vs. No OAC). Continuous variables are presented as mean ± standard deviation or median (interquartile range), according to each study’s reporting. Categorical variables are presented as number (percentage).

Randomis, randomization; M, months; Treat, treatment; AF, atrial fibrillation; Subj, subjects; Parox, paroxysmal; Persist, persistent; Long, longstanding; DOAC, direct oral anticoagulation; IQR, interquartile range; HF, heart failure; HTN, hypertension; DM, diabetes mellitus; LA, left atrial; LVEF, left ventricular ejection fraction; NR, not reported; TIA, transient ischaemic attack.

**Table 2:** Trial characteristics across the three randomized trials

Trial	Inclusion criteria	Definition of arrhythmia-free requirement at entry	OAC arm	Comparator arm	Rhythm monitoring after randomization	OAC reinitiation rule	Follow-up duration
**ALONE-AF**	Age 19–80 years; prior AF ablation within the previous 1–5 years; CHA_2_DS_2_VASc ≥1 in men or ≥2 in women; no documented AF recurrence in the year before enrolment	Successful prior AF ablation with no documented atrial arrhythmia for at least 12 months before screening, confirmed by at least one 24–72 h Holter ECGs	Continuation of DOAC: apixaban 5 mg BD in 78%, 2.5 mg BD in 5.3%; rivaroxaban 15 mg OD in 8.7%, 20 mg OD in 7.3%; edoxaban 60 mg OD in 0.7%	OAC discontinued; antiplatelet use was discouraged	Protocol-directed clinic follow-up with 12-lead ECG at each visit and at least one 24–72-h Holter during follow-up; additional Holter or event monitoring and ECGs when symptoms suggested recurrence	If AF, atrial flutter, or atrial tachycardia was documented after randomization, OAC was restarted or initiated according to stroke risk	24 months
**OCEAN**	Adults who had undergone catheter ablation for AF at least 1 year earlier; deemed to have successful ablation; CHA_2_DS_2_VASc ≥1 in men or ≥2 in women	No clinical evidence of AF and no atrial tachyarrhythmia lasting ≥30 s on ECG and on at least one 24-h Holter between 2–6 months after ablation and another Holter performed at least 6 months after ablation	Rivaroxaban 15 mg once daily	Aspirin 70–120 mg once daily	In-person study visits at 1 and 3 months, then every 6 months; 12-lead ECG at each visit	If AF recurred or another clear indication for anticoagulation developed, investigators could switch or restart OAC	36 months
**ODIn-AF**	Adults undergoing catheter ablation for AF; CHA_2_DS_2_VASc score ≥ 2; Patients free from AF episodes 6 months after successful antral PVI	No documented AF/atrial tachyarrhythmia lasting ≥30 s during the 6-month post-ablation run-in, confirmed by ECG and 72-h Holter at the time of randomization.	Continuation of dabigatran 150 mg twice daily (with 110 mg twice daily for older or high-bleeding-risk patients)	Dabigatran stopped entirely; no routine antiplatelet therapy mandated	Systematic protocol-directed visits at 3, 9, and 12 months, at randomization, and at each visit: 12-lead ECG + 72-h Holter; additional ECG/Holter if symptomatic	Any documented AF recurrence or atrial tachyarrhythmia ≥30 s on ECG or Holter or requirement for cardioversion or repeat ablation	12 months

Inclusion criteria, definitions of arrhythmia-free status at randomization, treatment strategies, rhythm monitoring protocols, anticoagulation reinitiation rules, and planned follow-up duration for the ALONE-AF, OCEAN, and ODIn-AF trials. Information is presented as described in each protocol or primary publication.

AF, atrial fibrillation; DOAC, direct oral anticoagulant; ECG, electrocardiogram; OAC, oral anticoagulation; PVI, pulmonary vein isolation

The *Anticoagulation Alone After Atrial Fibrillation Ablation study* (ALONE-AF) compared continued direct oral anticoagulant therapy with discontinuation after at least 1 year (median of 29 months) without recurrent atrial arrhythmia.^15^ The *Optimal Anticoagulation for Enhanced Risk Patients Post Catheter Ablation for Atrial Fibrillation study* (OCEAN) enrolled patients who had remained arrhythmia-free for a median of 16 months and randomized them to continuing rivaroxaban or switching to aspirin.^16^ The *Oral Dabigatran After Pulmonary Vein Isolation in Atrial Fibrillation study* (ODIn-AF) included patients who had remained free of AF for 6 months following pulmonary vein isolation and assigned them to continue or discontinue dabigatran.^17^ The median duration of time from last ablation to discontinuation of OAC was not available for ODIn-AF.

Oral anticoagulation regimen differed across the three trials: dabigatran 110–150 mg BD was used for ODIn-AF,^17^ rivaroxaban 15 mg OD was used in OCEAN,^16^ and apixaban 2.5–5 mg BD, rivaroxaban 15–20 mg OD, or edoxaban 60 mg OD were used in ALONE-AF^15^ (*Table 2*).

Across the three studies, all participants had documented freedom from atrial arrhythmia confirmed by scheduled ECG or Holter monitoring prior to randomization. The mean CHA_2_DS_2_-VASc score ranged from 2 to 2.6, and the mean left ventricular ejection fraction was preserved in all cohorts (57–68%). The planned follow-up duration varied across the three studies, with an estimated median follow-up of 25.14 months (IQR 17.44–30.57 months). All studies used direct oral anticoagulants during the treatment phase. All three trials were judged to have an overall low risk of bias on the RoB 2 tool (see Supplementary material online, *Figure S1*). Each study was rated as having some concerns in the ‘deviations from intended interventions’ domain, primarily because all trials had an open-label design. No domains were rated as high risk and all other domains were judged to be at low risk of bias.

**Figure 2:**
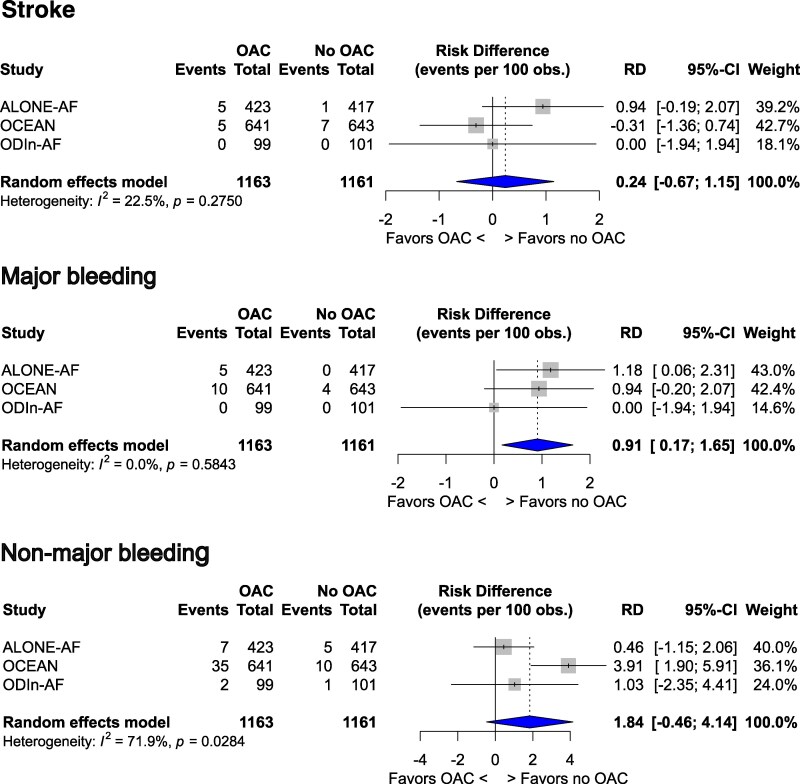
Risk differences for major clinical outcomes after discontinuation vs. continuation of oral anticoagulation following successful atrial fibrillation ablation. Each panel displays the pooled risk difference per 100 patients with 95% confidence intervals for the following outcomes: stroke, major bleeding, and nonmajor bleeding. Negative values favour OAC, and positive values favour no OAC. *CI,*  *confidence interval; OAC, oral anticoagulation; RD,*  *risk difference.*

A summary of the pooled analyses is presented in *Table 3*. Across the three randomized trials, discontinuation of OAC after successful AF ablation was not associated with an increased risk of stroke compared with continued therapy: OAC 0.86% vs. no OAC 0.69%; RD 0.24%; 95% CI −0.67% to 1.15%; *P* = 0.61; I^2^ = 22.5% (*Figure 2*). When standardized for follow-up duration, the pooled IRR of stroke occurrence was similarly nonsignificant (IRR 1.32; 95% CI 0.32–5.41; *P* = 0.70; I^2^ = 17.1%), confirming no time-adjusted difference in annualized event incidence between groups (see Supplementary material online, *Figure S2*). No systemic embolic events were reported in either arm (*Table 3*). Silent (asymptomatic) stroke detected on follow-up MRI also showed no difference between groups (OAC 0.27% vs. no OAC 0.27%; RD 0.24%; 95% CI −1.70% to 2.19%); *P* = 0.25; I^2^ = 45%) (see Supplementary material online, *Figure S2*).

**Table 3:** Summary of findings

Outcome	Number of trials	OAC event rate	No OAC event rate	Risk difference *P-*value	NNH/NNT	Heterogeneity and RoB assessment	Indirectness imprecision Publication bias	Interpretation Quality of evidence/GRADE
**All strokes**	3	10/1163	8/1161	+0.24% (95% CI −0.67 to 1.15) *P* = 0.61	417 (95% CI 87 to –149)	Low heterogeneity (I^2^ = 22.5%) RoB – ↓1 level (Performance)	No No NA	Moderate ⊕⊕⊕⊖
**Systemic embolism**	3	0/1163	0/1161	—	—	—	—	—
**Major bleeding**	3	15/1163	4/1161	+0.91% (95% CI 0.17 to 1.65) *P* = 0.02	110 (95% CI 61 to 588)	Low heterogeneity (I^2^ = 0%) RoB – ↓1 level (Performance)	No No NA	Moderate ⊕⊕⊕⊖
**Silent (asymptomatic) Stroke**	2	2/740	2/744	+0.24% (95% CI -1.70% to 2.19%) *P* = 0.25	417 (95% CI –59 to 46)	Moderate heterogeneity (I^2^ = 45%) RoB – ↓1 level (Performance)	No No NA	Moderate ⊕⊕⊕⊖
**Nonmajor bleeding**	3	44/1163	16/1161	+1.84% (95% CI −0.46 to 4.14) *P* = 0.12	54 (95% CI 24 to –217)	Considerable heterogeneity (I^2^ = 71.9%) RoB – ↓1 level (Performance)	No No NA	Moderate ⊕⊕⊕⊖
**Gastrointestinal bleeding**	3	5/1163	2/1161	+0.27% (95% CI −0.23 to 0.77) *P* = 0.29	370 (95% CI 130 to –435)	Low heterogeneity (I^2^ = 0%) RoB – ↓1 level (Performance)	No No NA	Moderate ⊕⊕⊕⊖
**Intracerebral haemorrhage**	3	7/1163	1/1161	+0.51% (95% CI −0.01 to 1.04) *P* = 0.06	196 (95% CI 96 to –10 000)	Low heterogeneity (I^2^ = 0%) RoB – ↓1 level (Performance)	No No NA	Moderate ⊕⊕⊕⊖

NNT/NNH values are calculated as the inverse of the absolute risk difference; confidence intervals are derived from the reciprocal of the 95% CI limits of the risk difference. Heterogeneity is expressed as I^2^ with qualitative interpretation according to Cochrane thresholds. Risk of bias and GRADE assessments indicate the overall certainty and reliability of each pooled estimate. Note: GRADE assessment downgraded to moderate for all outcomes due to risk of bias (performance bias).

AF, atrial fibrillation; CI, confidence interval; OAC, oral anticoagulant; GRADE, Grading of Recommendations Assessment, Development and Evaluation; I^2^, inconsistency statistic; NNH, number needed to harm; NNT, number needed to treat; RD, risk difference; RoB, risk of bias.

Continuing anticoagulation was associated with a significantly higher rate of major bleeding (OAC 0.90% vs. no OAC 0.34%; RD, 0.91%; 95% CI 0.17–1.65%; *P* = 0.02; I^2^ = 0%), corresponding to a number needed to harm of 110 (1 additional major bleeding per 110 patients) (*Figure 2*). Nonmajor bleeding also occurred more frequently among patients who remained on anticoagulation, although this difference did not reach statistical significance (RD 1.84%; 95% CI −0.46% to 4.14%; *P* = 0.12; I^2^ = 71.9%) (*Figure 2*).

Gastrointestinal bleeding was numerically more common with continued anticoagulation but did not reach significance (RD 0.27%; 95% CI −0.23% to 0.77%; *P* = 0.29; I^2^ = 0%) (see Supplementary material online, *Figure S4*). Intracerebral haemorrhage demonstrated a similar trend towards higher incidence in the continued anticoagulation group (RD 0.51%; 95% CI −0.01% to 1.04%; *P* = 0.06; I^2^ = 0%) (see Supplementary material online, *Figure S5*).

**Figure 3:**
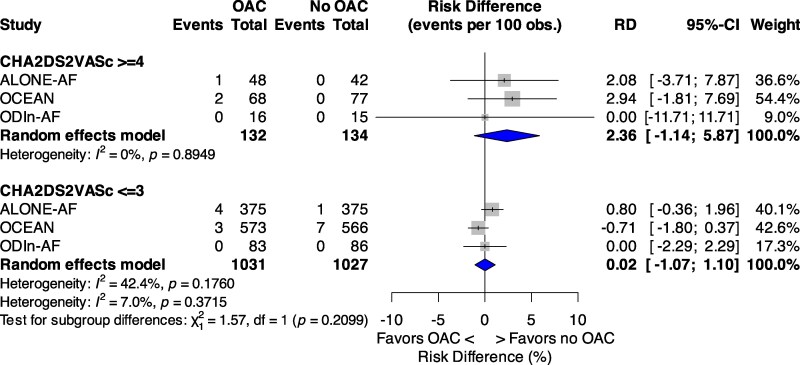
Risk differences for stroke after discontinuation vs. continuation of oral anticoagulation following successful atrial fibrillation ablation: subanalysis for high and low CHA_2_DS_2_VASc scores. Each panel displays the pooled risk difference per 100 patients with 95% confidence intervals for the outcome of stroke. Negative values favour OAC, and positive values favour no OAC. *CI,*  *confidence interval; OAC, oral anticoagulation; RD,*  *risk difference.*

In a subgroup analysis of patients with high CHA_2_DS_2_VASc scores (≥4), stroke occurred in 3 of 132 patients (2.3%) who continued OAC and in 0 of 134 patients (0%) who discontinued. The pooled RD was 2.36% (95% CI −1.14% to 5.87%), not significant (*P* = 0.19) (*Figure 3*). For patients with a CHA_2_DS_2_VASc score of ≤3, no significant differences were observed either: stroke occurred in 7 of 1031 patients who continued OAC and in 8 of 1027 of those that discontinued OAC (RD 0.02, 95% CI −1.07% to 1.10%, *P* = 0.98, I^2^ = 42%). There was no significant difference between groups (*P* = 0.21).

In sensitivity analyses using relative risk models, results were consistent with the primary analysis. Discontinuation of anticoagulation was not associated with an increased relative risk of stroke (RR 1.51; 95% CI 0.24–9.47; *P* = 0.66). Continuing anticoagulation remained associated with higher relative risks of major bleeding (RR, 3.07; 95% CI 1.05–8.96; *P* = 0.04) and nonmajor bleeding (RR 2.49; 95% CI 1.19–5.22; *P* = 0.02) (see Supplementary material online, *Table S3*).

We were unable to perform meta-regressions or generate funnel plots because an insufficient number of randomized controlled trials met the inclusion criteria. No data were available for other subgroup analyses.

## Discussion

In this meta-analysis of three recent randomized trials including 2324 low to moderate thromboembolic risk patients who remained arrhythmia-free after catheter ablation, discontinuation of OAC did not increase the risk of stroke or systemic embolism compared with continued therapy. Absolute thromboembolic event rates were low in both groups. By contrast, continued anticoagulation was associated with a significant excess of major bleeding and a numerical excess of nonmajor, gastrointestinal, and intracranial bleeding. Taken together, these findings suggest that in carefully selected post-ablation patients with maintained sinus rhythm, the net clinical benefit of long-term anticoagulation is attenuated and may favour treatment withdrawal. Current guidelines recommend that anticoagulation after AF ablation be continued and that longer term therapy be guided by baseline CHA_2_DS_2_VASc score rather than apparent rhythm status, reflecting concerns regarding silent AF recurrence.^2^ Large real-world registry analyses have reported a bleeding–thromboembolism trade-off following OAC discontinuation. For example, a recent nationwide Japanese electronic health record study of over 200 000 patients undergoing AF ablation found that stopping anticoagulation reduced bleeding, but thromboembolic risk remained elevated in patients with high CHADS_2_ scores, whereas those with lower baseline risk had very low absolute stroke rates off anticoagulation.^20^ A recent cohort study suggested that risk of thromboembolic events in patients discontinuing OAC is higher among patients with asymptomatic AF, LV ejection fraction of <60%, and LA diameter ≥45 mm.^21^ However, the nonrandomized nature of these studies, clinician-directed anticoagulation, lack of protocolized rhythm monitoring, and absence of systematic silent cerebral infarction assessment preclude a conclusive answer on OAC withdrawal in high-risk patients. Consequently, further randomized controlled trials are required to address this knowledge gap.

The interpretation of these findings must be guided by the characteristics of the enrolled patients. Across the three randomized trials (ODIn-AF, ALONE-AF, and OCEAN), the study populations were largely composed of individuals with low to moderate thromboembolic risk. ODIn-AF required a CHA_2_DS_2_VASc score of at least 2 and a mean score of 2.6.^17^ Previous stroke or transient ischaemic attack was an exclusion criterion, and few participants had scores of 5 or more. ALONE-AF enrolled men with a CHA_2_DS_2_VASc score of at least 1 and women with a score of at least 2.^15^ OCEAN AF had a mean score of 2.2, with approximately one-third having scores of 3 or more. This profile differs from the previous nonrandomized data coming from large registries and cohort studies where older age, prior stroke, heart failure, and structural atrial disease were more frequent.^17^ Whilst some of this early nonrandomized evidence described comparable stroke rates off anticoagulation with substantially less bleeding, some studies evaluating early discontinuation or including high-risk thromboembolic risk patients often suggested an increase in ischaemic events.^7,21^ Importantly, a recent meta-analysis of 32 studies by Barbosa *et al.*^22^ found that stopping OAC significantly reduced bleeding but may increase thromboembolic events in patients with CHA_2_DS_2_VASc >2. However, that subanalysis was largely driven by nonrandomized data, which accounted for 95% of the analysis weight, and did not include data from ALONE-AF or ODIn-AF. In contrast, our meta-analysis included only randomized controlled trials, incorporated previously unpublished subgroup data, and focused on a more narrowly selected, lower thromboembolic risk patient population.

The pattern observed in this study is consistent with evidence from contemporary trials in low burden or subclinical AF. In NOAH AFNET 6, edoxaban did not reduce stroke or systemic embolism in patients with atrial high-rate episodes and a median CHA_2_DS_2_VASc score of 4, but it was associated with increased bleeding.^23^ Patients who remain in sinus rhythm after ablation appear to fall within a similar domain of low thromboembolic risk, in which the marginal prevention benefit of uninterrupted anticoagulation may be small and readily outweighed by bleeding.

The value of starting antiplatelet therapy after stopping OAC remains uncertain. In OCEAN, rivaroxaban did not reduce clinical or covert stroke compared with aspirin, and bleeding was higher with rivaroxaban, yet aspirin itself did not provide any clear protection in patients who were already arrhythmia-free.^16^ In the other two trials in our meta-analysis, patients who stopped OAC were not routinely given aspirin or other antiplatelet agents, and stroke rates still remained extremely low. Evidence from broader AF trials shows a similar pattern; aspirin offers only modest or no stroke prevention but still increases bleeding risk, as seen in AVERROES (major bleeding 1.2% per year with aspirin) and ACTIVE A/W trials, where antiplatelet therapy delivered far less protection than anticoagulation.^24,25^ Overall, the evidence points to a minimal role for antiplatelet therapy following ablation in patients who lack other indications for antiplatelet treatment.

The findings also help clarify the appropriate timing for considering withdrawal of anticoagulation. Current European and American guidelines recommend anticoagulation for at least 2–3 months after ablation and long-term therapy thereafter according to the CHA_2_DS_2_VASc score, irrespective of procedural success.^8,9^ These recommendations were published prior to availability of randomized evidence and were influenced by the well-recognized problem of atrial arrhythmia recurrence. In the present trial, randomization took place only after a substantial period without documented arrhythmia. This occurred at 6 months or later in ODIn AF and at 12 months or later in ALONE-AF and OCEAN. Event rates remained below 1% per year regardless of whether anticoagulation was continued or discontinued. These observations support the potential role of AF ablation in reducing the risk of stroke that has been demonstrated by two recent systematic reviews of randomized controlled trials^5,6^ and support a landmark approach in which withdrawal is considered only after an extended period of rhythm stability rather than during the early inflammatory interval after ablation.

Withdrawal of anticoagulation should only be contemplated when recurrence of AF can be detected reliably. Asymptomatic episodes are common after ablation, and symptom-based follow-up is inadequate. The trials used a structured rhythm surveillance strategy that included frequently scheduled electrocardiograms or short-duration Holter monitoring, supplemented by symptom-prompted recordings. Although intermittent monitoring can inevitably miss some short or low burden episodes, the consistently low stroke rates observed in both treatment groups suggest that undetected recurrences were not associated with a material rise in thromboembolic risk in the populations studied.^26,27^ This level of monitoring serves as a practical approach, as continuous invasive monitoring is limited by cost and invasiveness and is not routinely indicated in clinical practice. Any decision to stop anticoagulation should therefore be accompanied by a clear plan for rhythm assessment using periodic electrocardiography, Holter monitoring, or validated wearable technologies. A recent analysis of the CIRCA-DOSE trial suggests that monitoring using wearable technology outperforms conventional noninvasive strategies (e.g. Holter monitors) for recurrence detection and strongly correlates with implantable cardiac monitor-derived AF burden.^28,29^

The overall evidence supports a more individualized strategy rather than a universal expectation of lifelong anticoagulation in post-ablation patients who meet CHA_2_DS_2_VASc criteria. In this meta-analysis, most participants had low CHA_2_DS_2_VASc scores. The combined evidence suggests that in patients who remain free of arrhythmia after ablation and are at low thromboembolic risk, particularly CHA_2_DS_2_VASc 0–2 in men or 1–3 in women, withdrawal may be reasonable when combined with an organized plan for close arrhythmia surveillance. In contrast, for patients with higher scores, specifically CHA_2_DS_2_VASc ≥4, the evidence remains uncertain. Given their elevated baseline stroke risk, maintaining anticoagulation may be the more prudent approach until further data regarding these high-risk groups becomes available.

Important limitations with this review are to be recognized. Only three randomized controlled trials were included. Variations in trial design such as OAC regimens, study populations, control group (aspirin vs. no therapy), and follow-up protocol limit the wider applicability of the pooled results. The number of stroke and systemic embolism events was small, which leads to wide CIs (i.e. imprecision) and limits the ability to exclude modest differences in risk. Definitions of successful ablation and the intensity of rhythm monitoring varied between trials, and asymptomatic recurrences are likely to have been under-detected. Elderly patients and those with very high CHA_2_DS_2_VASc scores or significant ventricular dysfunction are underrepresented, which limits the generalizability of these findings. Follow-up in the trials did not extend beyond 3 years, so the long-term balance between stroke prevention and bleeding risk is yet to be determined.

## Conclusion

In patients who remain arrhythmia-free after AF ablation, discontinuing OAC did not increase stroke or systemic embolism and clearly reduced major bleeding. These findings suggest that lifelong anticoagulation after ablation may not be necessary for all patients. Provided that regular ECG monitoring is maintained, anticoagulation may be safely withdrawn in appropriately selected patients with low thromboembolic risk. However, given the limited number of randomized trials in this space, these findings require confirmation in larger studies.

## Supplementary Material

oeag067_Supplementary_Data

## Data Availability

No new data were generated or analysed in support of this research. All data were extracted from publications or provided by study authors and made available on the manuscript.
