# Supplementary material for: Oral anticoagulation discontinuation after atrial fibrillation ablation: a systematic review and meta-analysis of randomized trials
Source: Eur Heart J Open. 2026 May 22;6(3):oeag067. doi: 10.1093/ehjopen/oeag067 (PMC13252450; doi:10.1093/ehjopen/oeag067)

**Supplementary appendix**

**Supplementary table 1 search strategy**

| **Search Terms** |
| --- |
| ("atrial fibrillation" OR AF OR "paroxysmal atrial fibrillation" OR "persistent atrial fibrillation") **AND** ("catheter ablation" OR "radiofrequency ablation" OR "pulmonary vein isolation") **AND** (anticoagulant* OR "direct oral anticoagulant*" OR DOAC OR "vitamin K antagonist" OR antithrombotic* OR "stop anticoagulation" OR antiplatelet* OR cessation OR discontinue*) **AND** ("randomized controlled trial" OR trial) |

**Supplementary Table 2: Excluded studies**

| **Study title** | **Year** | **Source** | **Reason for exclusion** |
| --- | --- | --- | --- |
| No Effect of Continued Antiarrhythmic Drug Treatment on Top of Optimized Pulmonary Vein Isolation in Patients With Persistent Atrial Fibrillation: Results From the POWDER-AF2 Trial. | 2023 | https://dx.doi.org/10.1161/CIRCEP.123.012043 | Wrong intervention - no off-OAC arm |
| Anticoagulation, therapy of concomitant conditions, and early rhythm control therapy: a detailed analysis of treatment patterns in the EAST - AFNET 4 trial. | 2022 | https://dx.doi.org/10.1093/europace/euab200 | Wrong intervention - no off-OAC arm |
| Antithrombotic Treatment after Atrial Fibrillation Ablation. | 2020 | https://dx.doi.org/10.2174/1381612826666200407154329 | Wrong study design - not an RCT |
| Feasibility of Uninterrupted Direct Oral Anticoagulants with Temporary Switching to Dabigatran ("Dabigatran Bridge") for Catheter Ablation of Atrial Fibrillation. | 2019 | https://dx.doi.org/10.1536/ihj.19-143 | Wrong study design - not an RCT |
| PulmOnary vein isolation With vs. without continued antiarrhythmic Drug trEatment in subjects with Recurrent Atrial Fibrillation (POWDER AF): results from a multicentre randomized trial. | 2018 | https://dx.doi.org/10.1093/eurheartj/ehx666 | Wrong intervention - no off-OAC arm |
| Dual antiplatelet therapy versus warfarin anticoagulation in patients undergoing catheter ablation of atrial fibrillation. | 2014 | https://dx.doi.org/10.1016/j.ijcard.2014.03.027 | Wrong study design - not an RCT |
| The risk of thromboembolism and need for oral anticoagulation after successful atrial fibrillation ablation. | 2010 | https://dx.doi.org/10.1016/j.jacc.2009.11.039 | Wrong study design - not an RCT |
| Interruption of oral anticoagulants after atrial fibrillation catheter ablation: the importance of risk stratification | 2024 | https://dx.doi.org/10.1093/eurheartj/ehad874 | Editorial |
| Safety and efficacy of oral anticoagulation discontinuation in high thromboembolic risk patients at long term follow-up after successful atrial fibrillation ablation | 2019 | https://dx.doi.org/10.1093/eurheartj/ehz748.1143 | Wrong study design - not an RCT |
| Discontinuation of Oral Anticoagulation After Successful Atrial Fibrillation Ablation | 2025 | 10.1001/jamanetworkopen.2025.1320 | Wrong study design - not an RCT |
| Discontinuation of anticoagulants after successful surgical ablation of atrial fibrillation | 2020 | 10.1111/jocs.14719 | Wrong study design - not an RCT |
| Left atrial appendage occlusion in patients with nonvalvular atrial fibrillation: Present evidence, ongoing studies, open questions | 2020 | 10.1007/s00063-018-0500-4 | Wrong study design - not an RCT |

**Supplementary Table 3: Risk ratio sensitivity analysis**

| **Outcome** | **Number of studies** | **Event rate in OAC arm** | **Event rate in no OAC arm** | **Risk ratio** | **P value** | **I^2^ value** |
| --- | --- | --- | --- | --- | --- | --- |
| **All Strokes** | 2 | 10/1064 | 8/1060 | 1.51 (95% CI 0.24 to 9.47) | 0.66 | 58.7% |
| **Systemic Embolism** | 3 | 0/1163 | 0/1161 |  |  |  |
| **Major Bleeding** | 2 | 15/1064 | 4/1060 | 3.07 (95% CI 1.05 to 8.96) | 0.04 | 0% |
| **Silent (asymptomatic) Stroke** | 2 | 2/740 | 2/744 | 1.01 (95% CI 0.04 to 24.17) | 0.99 | 54.4% |
| **Non-major Bleeding** | 3 | 44/1163 | 16/1161 | 2.49 (95% CI 1.19 to 5.22) | 0.02 | 0% |
| **Gastrointestinal Bleeding** | 2 | 5/1064 | 2/1060 | 2.04 (95% CI 0.44 to 9.52) | 0.36 | 0% |
| **Intracerebral Haemorrhage** | 2 | 7/1064 | 1/1060 | 4.99 (95% CI 0.87 to 28.72) | 0.07 | 0% |

**Supplementary Figure 1: Risk of Bias using ROB 2.0 tool**

**Supplementary Figure 2: Pooled incidence rate ratio of stroke after OAC discontinuation versus continuation, standardised for follow-up duration**

**
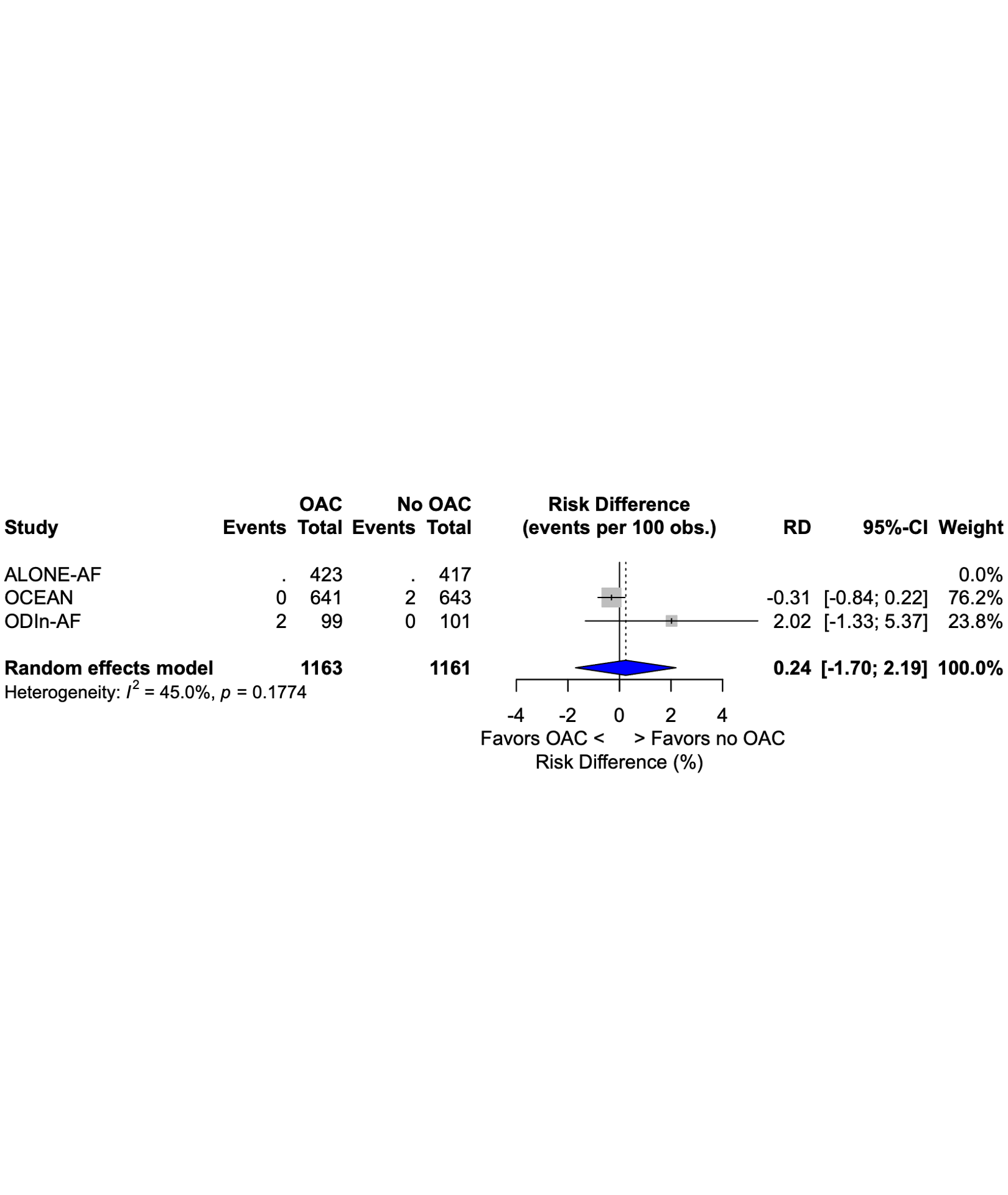
Supplementary Figure 3: Risk difference for silent stroke comparing OAC continuation vs discontinuation**

**Supplementary Figure 4: Risk difference for gastrointestinal bleeding comparing OAC continuation vs discontinuation**

**
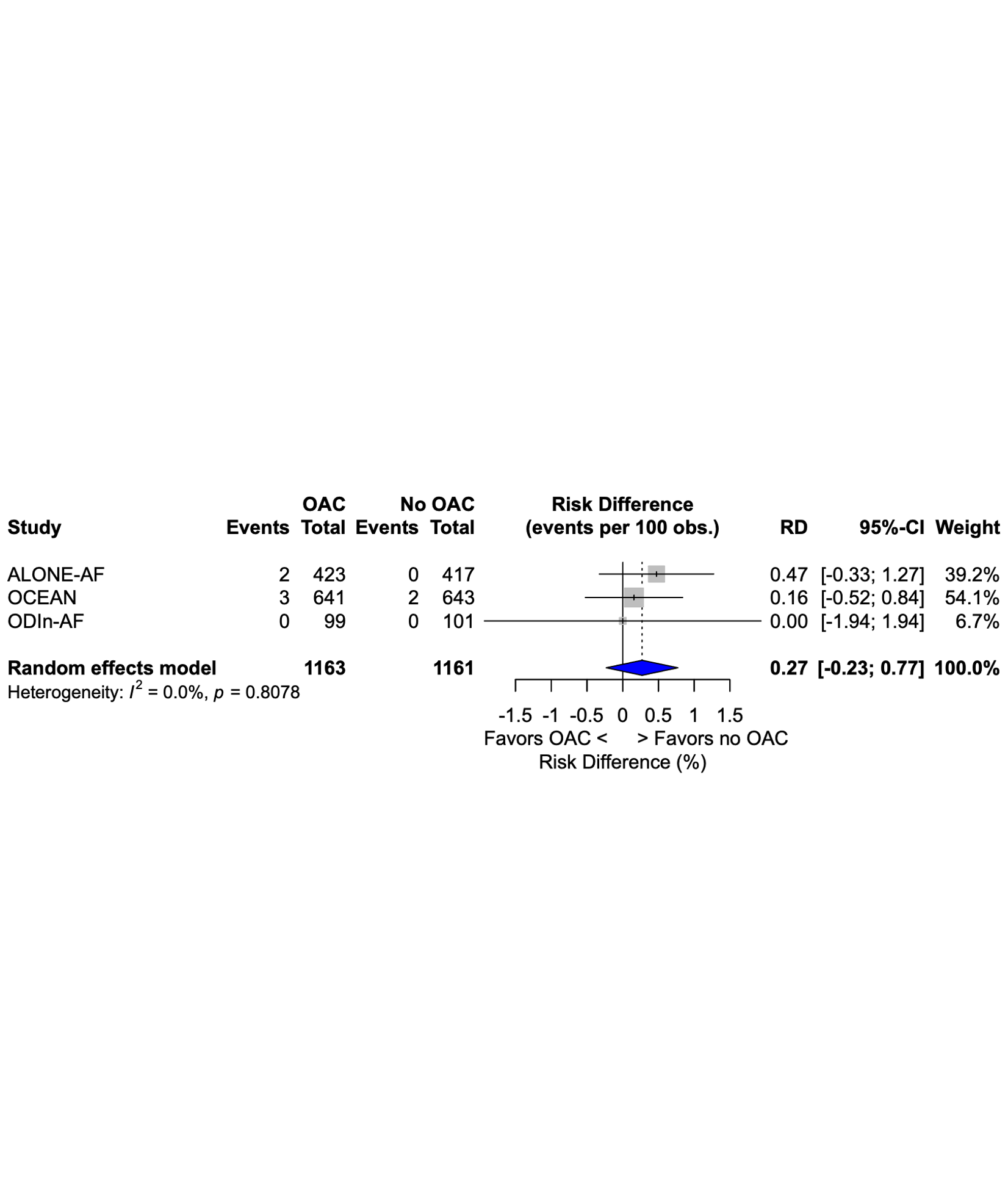
**

**Supplementary Figure 5: Risk difference for intracerebral haemorrhage comparing OAC continuation vs discontinuation**


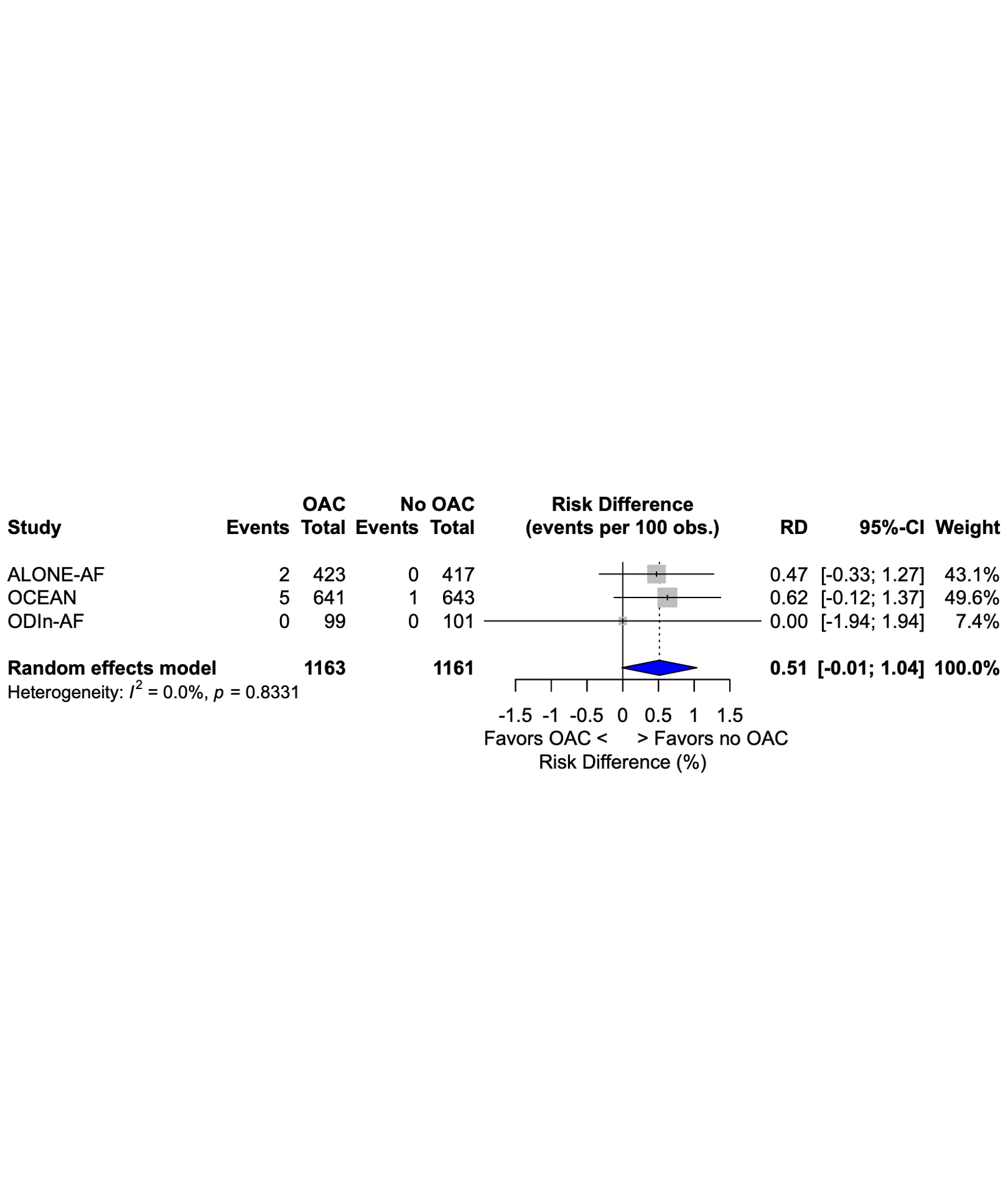

Supplement: oeag067_Supplementary_Data [file oeag067_supplementary_data.docx]
